# Immobilization of Lewis acidic ionic liquid on perlite nanoparticle surfaces as a highly efficient solid acid catalyst for the solvent-free synthesis of xanthene derivatives

**DOI:** 10.1039/c9ra03312b

**Published:** 2019-06-26

**Authors:** L. Moradi, M. Mirzaei

**Affiliations:** Department of Organic Chemistry, Faculty of Chemisry, University of Kashan P. O. Box 8731753153 Kashan Iran L_moradi@kashanu.ac.ir

## Abstract

In this study, perlite nanoparticles were prepared through a simple method and then modified with Lewis acidic ionic liquid (perlite NP@IL/ZrCl_4_) through a two step procedure. The prepared solid acid catalyst was characterized by Fourier transform infrared spectroscopy (FTIR), scanning electron microscopy (SEM), X-ray diffraction (XRD), energy-dispersive X-ray spectroscopy (EDX) and thermo gravimetric analysis (TGA). Perlite NP@IL/ZrCl_4_ was used as a new solid acid, reusable and green heterogeneous nanocatalyst for the one-pot synthesis of xanthene derivatives. Synthesis of xanthenes was performed under solvent free conditions using a catalytic amount (0.005 g, 0.4 mol%) of the prepared catalyst with simple work-up and high to excellent yield of products. The reusability and high efficiency of this catalyst makes this method attractive for large scale environment-friendly operations.

## Abstract 2

Perlite nanoparticles were prepared, modified with Lewis acidic ionic liquid, and used as a highly efficient catalyst for the eco-friendly, solvent free and high yield synthesis of xanthenes *via* a multicomponent reaction.

## Graphical abstract

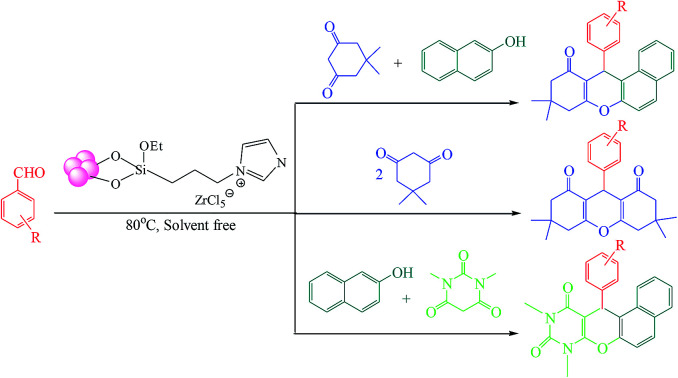

## Introduction

**Scheme 1:**
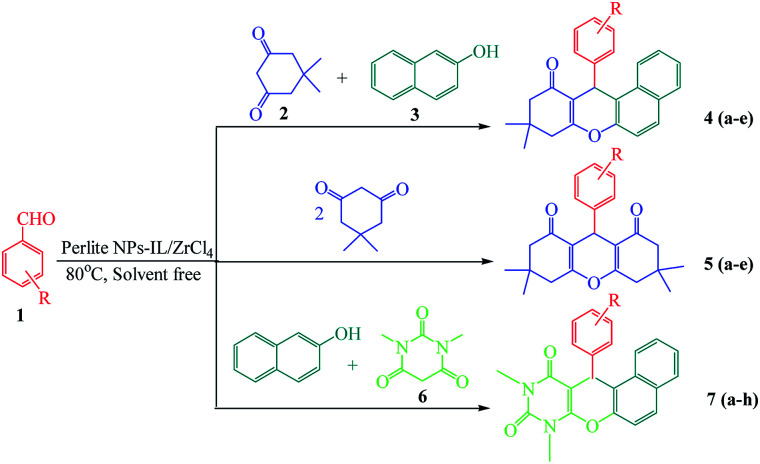
Solvent free one-pot synthesis of three types of xanthene derivatives in the presence of perlite NPs@IL/ZrCl_4_.

Xanthene and its derivatives are pharmaceutically and biologically active compounds.^1,2^ Because of the widespread use of this class of heterocyclic compounds in the preparation of dyes,^3^ laser technologies^4^ and pH-sensitive fluorescent materials for visualization of biomolecules,^5^ they have received significant attention in recent years. On the other hand, fused pyran ring systems especially pyranopyrimidines have biological activities including antimicrobial,^6^ anticonvulsant,^7^ antiplatelet,^8^ antibacterial,^9^ antiphlogistic,^10^ analgesic,^11^ anti-inflammatory,^11^ antigenotoxic^12^ and antifungal activities.^7,13–15^ These unique properties, led to intensive research on the synthesis of xanthene and naphthopyranopyrimidine derivatives in the presence of various catalysts such as CoPy_2_Cl_2_,^16^ I_2_,^17^ choline chloride,^18^ Y(NO_3_)_3_·6H_2_O and SnCl_2_·2H_2_O,^19^ ZnO NPs,^20^ Al(HSO_4_)_3_,^21^ sulfamic acid,^22^ perlite-SO_3_H,^23^ LiBr,^24^ silica sulfuric acid,^25^ (nBu)_4_NBr,^26^ TfOH,^27^ cellulose sulfamic acid,^28^ molecular iodine,^29^ heteropolyacid,^30^ phosphoric acid supported on alumina,^31^ [IMPS][TfO],^32^ [HNMP][HSO_4_],^33^ [TEBSA][HSO_4_],^34^ [MIMPS][HSO_4_],^35^ [DMEA][HSO_4_],^36^ [BDMAP][OH]^37^ and sulfonyl-functionalized acidic ionic liquids.^38^ In recent years, the application of supported catalysts was developed for the synthesis of organic compounds. In this way, using low cost and readily available supports has been considered for the preparation of this class of heterogeneous catalysts. Perlite is an amorphous volcanic glass containing SiO_2_ (70–75%), Al_2_O_3_ (12–15%), Na_2_O (3–4%), K_2_O (3–5%), Fe_2_O_3_ (0.5–2%), MgO (0.2–0.7%) and CaO (0.5–1.5%).^39,40^ Because of its low density and relatively low price, many commercial applications for perlite have been developed in the construction and manufacturing fields. It is used in lightweight plasters, concrete, insulation and ceiling tiles.^41^ In horticulture, perlite can be used as a soil amendment or alone as a medium for hydroponics or starting cuttings.^42^ Small quantities of perlite are also used in foundries, cryogenic insulation and in ceramics as a clay additive. It is also used by the explosives industry.^42^ It was found to be an excellent support for immobilization of catalysts and biocatalysts such as enzymes for bioremediation and sensing applications.^24,43,44^ To avoid of using the toxic organic solvents and due to the advantages of multicomponent reactions including low response time, high efficiency, clean work-up and easy purification, we have designed environmentally benign procedures for the one-pot synthesis of xanthene and naphthopyranopyrimidine derivatives under thermal and solvent free conditions using perlite NPS@IL/ZrCl_4_ as a new efficient and reusable solid acid catalyst (Scheme 1).

## Results and discussion

**Fig. 1:**
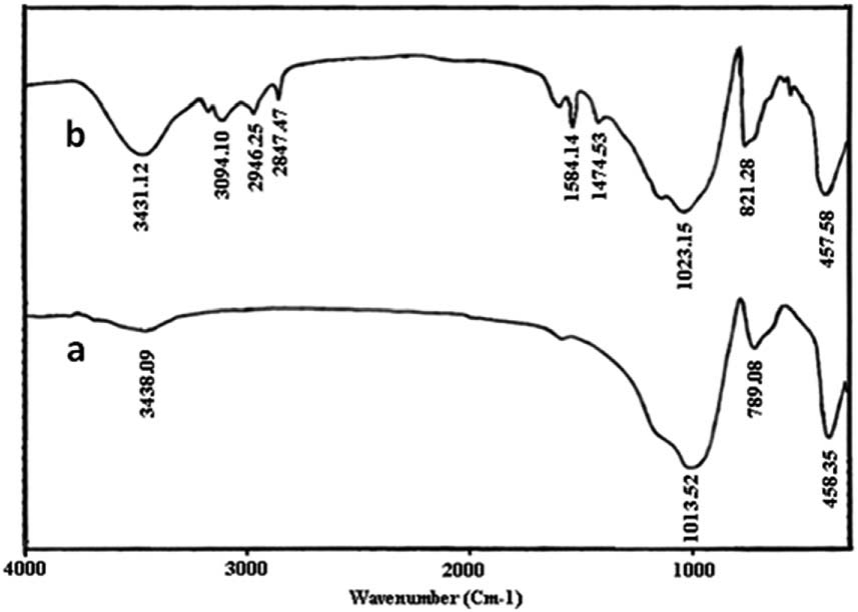
FT-IR spectra of (a) perlite NPs and (b) perlite NPs@IL/ZrCl_4_.

FT-IR spectra of perlite nanoparticles and perlite NPs@IL/ZrCl_4_ were presented in Fig. 1 to prove the catalyst structure. FTIR of perlite nanoparticles (Fig. 1a), shows the broad band at 3438.09 cm^−1^, correspond to O–H vibrations. Bands at 1013.52 and 789.08 cm^−1^ related to unsymmetrical and symmetrical Si–O–Si vibrations, respectively. The Si–O vibrations on perlite nanoparticles were appeared at 458.35 cm^−1^. In FTIR spectrum of perlite NPs@IL/ZrCl_4_, the band at 3431.12 cm^−1^ corresponds to O–H vibrations and stretching vibrations of =CH appeared at 3094.10 cm^−1^. Also the bands at 2946.25 and 2847.47 cm^−1^ are from –CH bond vibrations. Moreover, two bands at 1584.14 and 1474.53 cm^−1^ were existed from C=N and C=C vibrations, respectively. Stretching vibrations at 1023.15 and 821.28 cm^−1^ are related to unsymmetrical and symmetrical Si–O–Si vibrations. Finally, the Si–O absorbance recorded at 457.58 cm^−1^.

**Fig. 2:**
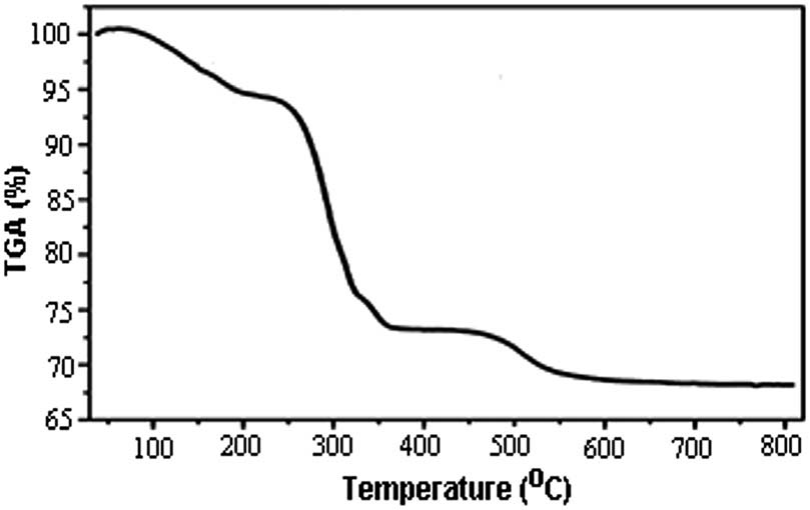
TGA analysis of perlite NPs IL/ZrCl_4_.

TGA curve of perlite NPs@IL/ZrCl_4_ at the range of 25 to 800 °C is shown in Fig. 2. The weight loss at the range of 100 to 200 °C was only 5%. This can be attributed to the removal of moisture from nanoparticle surfaces. A weight loss about 21% in the range of 300 to 350 °C is related to the separation of ionic liquid from the surface of catalyst. Finally, by removing the hydroxyl groups from perlite nanoparticle surfaces, a weight loss about 4% in the range of 480 to 520 °C is observed. Obtained results from TGA graph confirmed the chemical attachment of organic groups to the perlite NPs surfaces.

**Fig. 3:**
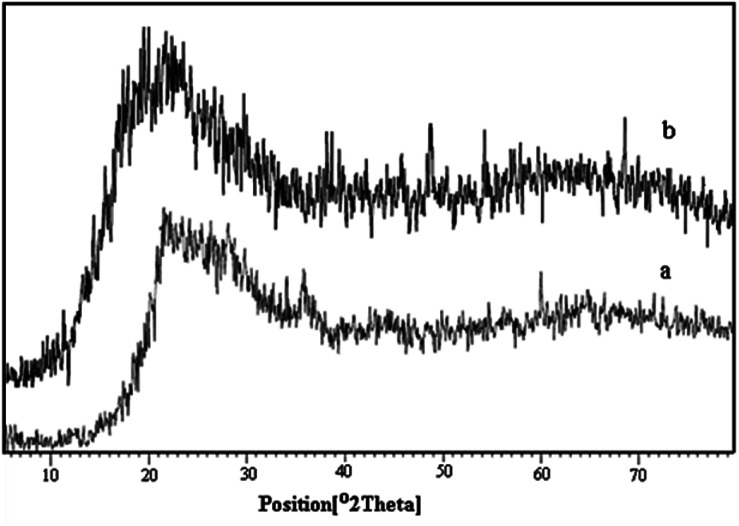
X-ray diffraction (XRD) pattern of (a) perlite and (b) perlite-IL/ZrCl_4_ nanoparticles.

X-ray diffraction analysis was used for investigating the structure of perlite nanoparticles and perlite NPs@IL/ZrCl_4_. The obtained diffractograms are displayed in Fig. 3. As shown in this Figure, XRD patterns of primary and modified perlite nanoparticles exhibit the same characteristic peaks which shows that the peaks and relative intensities match well with the X-ray diffraction pattern of amorphous particles and the structure of the primary perlite NPS preserved in the prepared catalyst. These graphs also show that the presence of organic species has no effect on the structure of perlite NPs. The peaks related to ZrCl_4_ are recorded at 2*θ* = 30, 33, 50.5 and 60°. In the XRD pattern of perlite NPs@IL/ZrCl_4_, the peaks of 2*θ* = 33 and 50.5° are distinguishable and two other peaks are covered by other broad peaks.

**Fig. 4:**
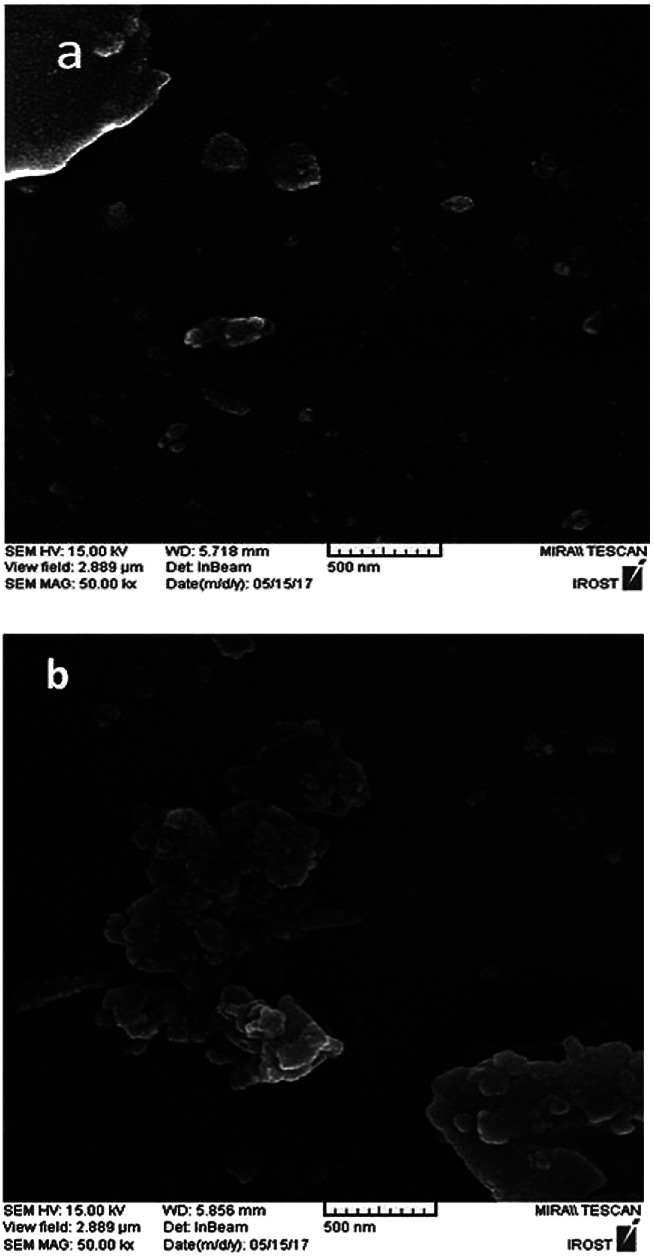
SEM photographs of (a) perlite NPs and (b) perlite NPs@IL/ZrCl_4_ nanoparticles.

The scanning electron microscope (SEM) images of the perlite NPs and perlite NPs@IL/ZrCl_4_ were displayed in Fig. 4(a and b). These images clearly show the nanosize structure of perlite NPs and modified sample. As can be seen from Fig. 4b, the morphology of the perlite NPs@IL/ZrCl_4_ was quite similar to the raw nanoparticles and there are no changes in nanoparticle morphology after modification process.

**Fig. 5:**
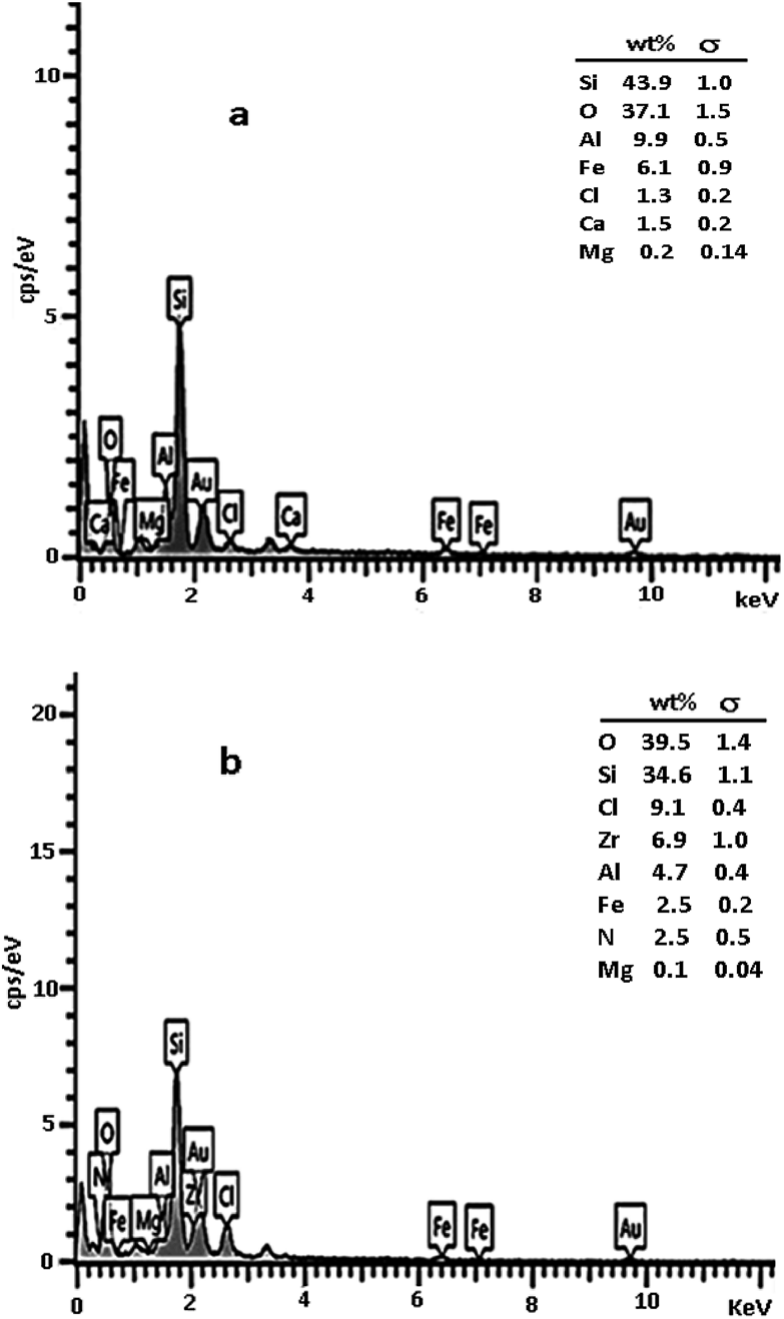
EDX of perlite NPs (a) and perlite NPS@IL/ZrCl_4_ (b).

The composition of perlite nanoparticles and synthesized catalyst was investigated by energy-dispersive X-ray spectroscopy (EDX). Results clearly confirmed the existence of Zr, Cl and N elements in the prepared catalyst structure (Fig. 5) and consequently proved that ionic liquid and ZrCl_4_ were attached successfully to the perlite nanoparticle surfaces.

As can be seen, the Zr and N content was 6.9% and 2.5% respectively. It concluded that the percentage of ionic liquid immobilized on perlite surfaces was about 1.25%. Further amount of Zr demonstrated the chelating of Zr to OH groups existed on the perlite surfaces (addition to be as zwitterion for imidazolium ion). Furthermore, the mol% of catalyst based on Zr ration was about 0.4 mol%.

After the preparation and characterization of catalyst, reaction conditions including the amount of catalyst and temperature were optimized. Firstly, optimum amount of catalyst was determined using the reaction between dimedone (2 mmol) and 3-nitrobenzaldehyde (1 mmol) in the presence of various amounts of catalyst at 80 °C. Results in Table 1 show that the best yield of product was obtained in the presence of 0.005 g (0.4 mol%) of catalyst (entry 2); also when the reaction was carried out in the absence of catalyst, the yield of product was only 5% after 100 min (entry 5).

**Table 1:** Effects of catalyst amounts on yield of 5a^a^

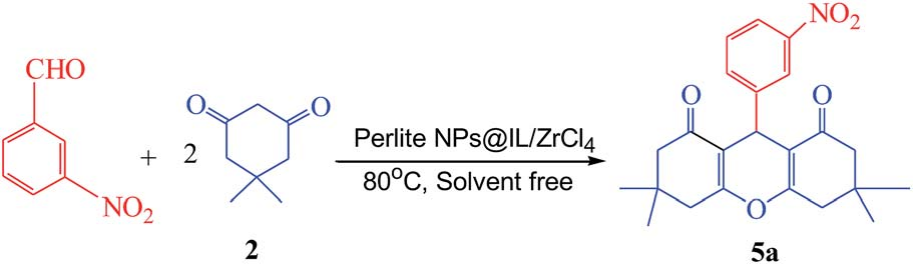			
Entry	Catalyst (g)	Time (min)	Yield (%)
1	0.003	140	85
2	0.005	100	92
3	0.008	100	92
4	0.01	85	92
5	—	100	5

a Isolated yield.

For evaluation the temperature effect on the yield of product, the model reaction was done in the presence of 0.005 g (0.4 mol%) of catalyst at different temperatures (Table 2). Obtained results show that 80 °C was the best temperature (entry 3). Furthermore, the yield of product at 100 °C was decreased due to decomposition of products after 100 min (entry 5).

**Table 2:** Optimization of reaction temperature^a^

Entry	*T* (°C)	Time (min)	Yield (%)
1	60	180	58
2	70	130	75
3	80	100	92
4	90	100	92
5	100	100	88

a Dimedone (2 mmol), 3-nitrobenzaldehyde (1 mmol) in the presence of 0.005 g (0.4 mol%) of catalyst.

After the determination of optimized conditions, appraisal of the catalyst efficiency was estimated using the reaction of a variety of aryl aldehydes, 2-naphthol and 1,3-dicarbonyl compounds such as dimedone and barbituric acid for preparation of xanthene derivatives in the presence of catalytic amount of perlite NPs@IL/ZrCl_4_. Obtained results in Table 3 show that aldehydes bearing electron withdrawing groups lead to products with higher yields after shorter times. Also in case of aldehydes with electron donating groups, it was observed that yield of reaction was lower after longer times.

**Table 3:** One pot synthesis of xanthene derivatives in the presence of perlite NPs@IL/ZrCl_4_ at 80 °C under solvent free conditions

Entry	R	Product	Time (min)	Yield (%)	MP (°C)
1	4-CH_3_	4a	110	91	206–208 (ref. 45)
2	4-OH	4b	120	89	215–217 (ref. 45)
3	4-OCH_3_	4c	115	88	204–206 (ref. 45)
4	2-Cl	4d	113	91	178–180 (ref. 45)
5^a^	4-CHO	4e	120	91	306–308 (ref. 45)
6	3-NO_2_	5a	100	92	171–173 (ref. 45)
7	2,4-Cl_2_	5b	115	89	245–247 (ref. 45)
8	4-CH_3_	5c	120	89	240–242 (ref. 45)
9	2-Cl	5d	112	91	225–227 (ref. 45)
10^b^	4-CHO	5e	120	91	228–230 (ref. 20)
11	4-OH	7a	115	91	286–288 (ref. 46)
12	2,4-Cl_2_	7b	113	88	263–265 (ref. 46)
13	4-NO_2_	7c	90	94	286–288 (ref. 46)
14	4-CH_3_	7d	130	88	195–197 (ref. 46)
15	4-OCH_3_	7e	133	83	292–294 (ref. 46)
16	2-OH	7f	120	90	288–290 (ref. 47)
17	3-OCH_3_	7g	105	92	283–285 (ref. 48)

a 2 (2 mmol), 3 (2 mmol), terephthaldehyde (1 mmol).

b Dimedone (4 mmol), aryl aldehyde (1 mmol).

**Scheme 2:**
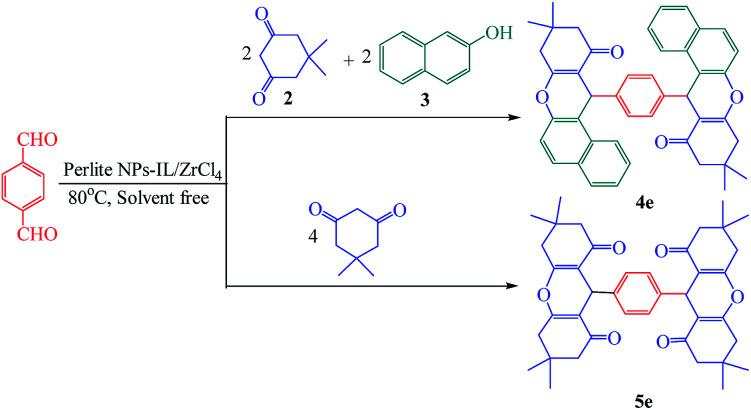
Preparation of dimeric xanthenes.

Further study was done using the reaction of terephthaldehyde with 2 mmol of dimedone (2) and 2 mmol of 2-naphthol (3) for the preparation of 4e. Symmetric dimer product was prepared with the reaction of terephthaldehyde with 4 mmol of dimedone (2) for preparation of 5e (Scheme 2). The yield of products was excellent and the spectroscopic data of these products demonstrated the structure of prepared compounds.

In continue, the efficiency of prepared catalyst was compared with some of other reported catalyst applied for the synthesis of 4c, 5a and 7c (as examples of three types of synthesized xanthene derivatives). The results are summarized in Table 4. As shown in this table, best yields were obtained using 0.005 g (0.4 mol%) of perlite-NPs@IL/ZrCl_4_ at 80 °C in the absence of solvent (Entry 6, 18 and 27). Catalyst amount in presented method is less than most of catalysts depicted in Table 4 demonstrated the high efficiency of solid acid catalyst.

**Table 4:** Compression between the efficiency of perlite-NPs@IL/ZrCl_4_ and some of other catalysts in synthesis of xanthenes and naphthopyranopyrimidines^a^

Entry	Catalyst	Time	Yield (%)	*T* (°C)	Ref.
1	[*n*-Pr_2_NH_2_][HSO_4_] (50 mol%)	15 min	82	80	49
2	BBSIC^b^ (2 ml)	10 min	80	50	50
3	P_2_O_5_ (20 mol%)	55 min	71	120	51
4	InCl_3_ (30 mol%)	45 min	76	120	51
5	TCCA^c^ (5 mol%)	40 min	78	110	45
6	Perlite@Il/ZrCl_4_ (0.005 g, 0.4 mol%)	115 min	88	80	—
7	Cu(NO_3_)_2_, 3H_2_O (10 mol%)	9.5 h	91	110	52
8	[BMIM]HSO_4_ (0.1 g)	3 h	87	80	53
9	[CMMIM]Cl, sonication (0.2 g)	1 h	87	rt	54
10	Co(HSO_4_)_2_ (0.025 g)	4 h	80	100	55
11	Nafion-H (0.485 g)	12 h	75	125	56
12	Choline peroxydisulfate (2 mmol)	5 min	88	105	57
13	TCCA (5 mol%)	20 min	88	110	45
14	TMGT^d^ (50 mmol)/TFA (60 mmol)	25 min	92	75	58
15	[Et_3_N–SO_3_H]Cl (15 mol%)	40 min	97	80	59
16	[Bmim][BF_4_]/Mg(BF_4_)_2_ (1 ml/0.5 mol%)	15 min	87	80	60
17	Perlite NPs@IL/ZrCl_4_ (0.005 g, 0.4 mol%)	100 min	92	80	—
18	Al(H_2_PO_4_)_3_ (0.1 g)	40 min,	80	110	61
19	ZrOCl_2_/nano TiO_2_ (3 mol%)	25 min	85	100	48
20	Heteropolyacid (5 mol%)	24 min	90	100	62
21	H_3_PO_4_/Al_2_O_3_ (0.1 g/50% w/w)	50 min	87	120	31
22	I_2_ (10 mol%)	55 min	86	120	63
23	InCl_3_ (35 mol%)	25 min	78	120	51
24	P_2_O_5_ (20 mol%)	80 min	58	120	51
25	SiO_2_@HClO_4_ (3 mol%)	1.5 h	94	125	64
26	Perlite@IL/ZrCl_4_ (0.005 g, 0.4 mol%)	90 min	94	80	—

a Entries 1–6 for solvent free preparation of 4c, 7–17 for preparation of 5a and 18–26 for 7c.

b 1,10-Butylenebis(3-sulfo-3*H*-imidazol-1-ium) chloride.

c Trichloroisocyanuric acid.

d 1,1,3,3-*N*,*N*,*N*′,*N*′-Tetramethylguanidinium trifluoroacetate.

On the other hand, in most reaction conditions (16 cases) the temperature is higher than 80 °C (applied temperature in presented method) and the yield of products is lower. As a result, Lewis acidic ionic liquid supported catalyst is more active than others in term of time, catalyst amount and temperature. In fact, Zr atoms (as Lewis acid sites) loaded in catalyst surfaces, can be activated the reactants (as shown in reaction mechanism) and catalyzed the solvent-free synthesis of xanthenes. Results collected in Table 4 shown that prepared catalyst improved the yield of products compare with other catalysts.

Furthermore, easy available and very low cost material of catalyst support (perlite), facile procedure for preparation of perlite nanoparticles as well as easy functionalization process, reusability and easy work up are some of the advantageous of proposed method. As can be seen in Table 4, most of catalysts are expensive, homogeneous, toxic and without facile availability.

Consequently, presented method has proved to be very effective, green, eco-friendly, safe and easy to operate and also the scale up of this method is easy.

**Scheme 3:**
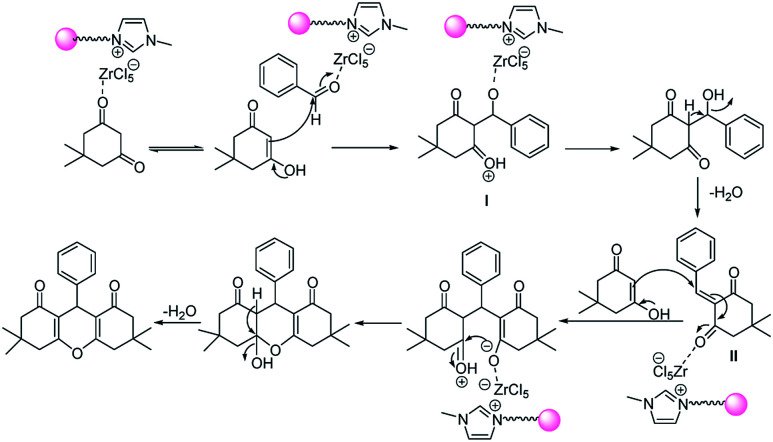
Suggested mechanism for the one-pot synthesis of xanthene derivatives in the presence of perlite NPs@IL/ZrCl_4_ nanoparticles.

Suggested mechanism for the synthesis of xanthene derivatives is shown in Scheme 3. As can be seen, in the initial step, Lewis acidic ionic liquid catalyzed the conversion of keto to enol form of 1,3 dicarbonyl; on the other hand, catalyst activated the carbonyl group of aldehyde and the nucleophilic addition of 1,3 dicarbonyl compound to activated aldehyde lead to formation of (I). Removal of H_2_O and nucleophilic addition of second mole of 1,3 dicarbonyl compound to I, created the intermediate II. Finally, cyclization and H_2_O removal from (II), give subsequent xanthene.

**Fig. 6:**
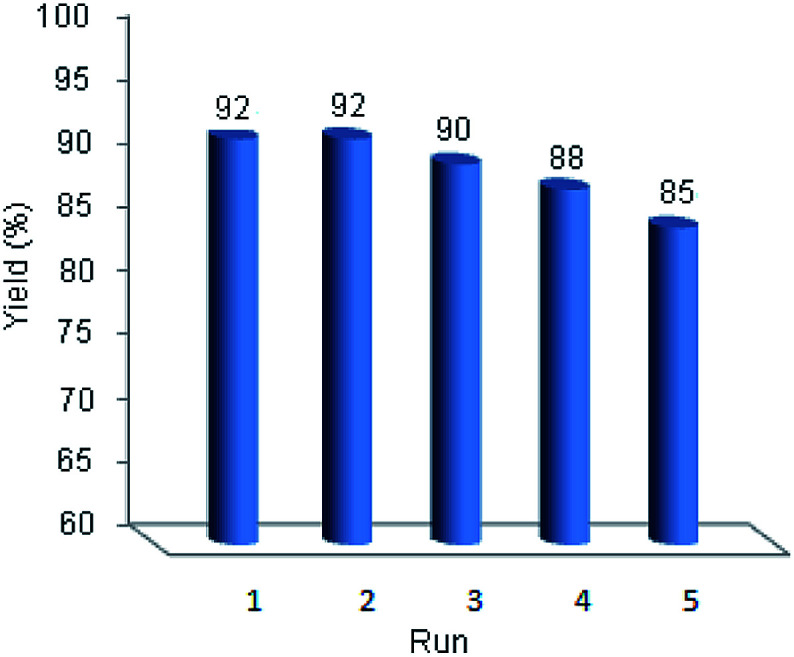
Reusability of perlite NPs@Il/ZrCl_4_.

One of the outstanding advantages of the catalysts is their reusability and stability which makes them valuable for commercial applications. Study on the reusability of prepared catalyst was done using model reaction. After completion of the reaction, the catalyst was separated. The recovered catalyst was washed with chloroform (3 × 5 ml) and dried to use for the next run in current reaction under equal conditions. It was found that the catalyst could be reused for four times without considerable loss of its activity (Fig. 6).

**Fig. 7:**
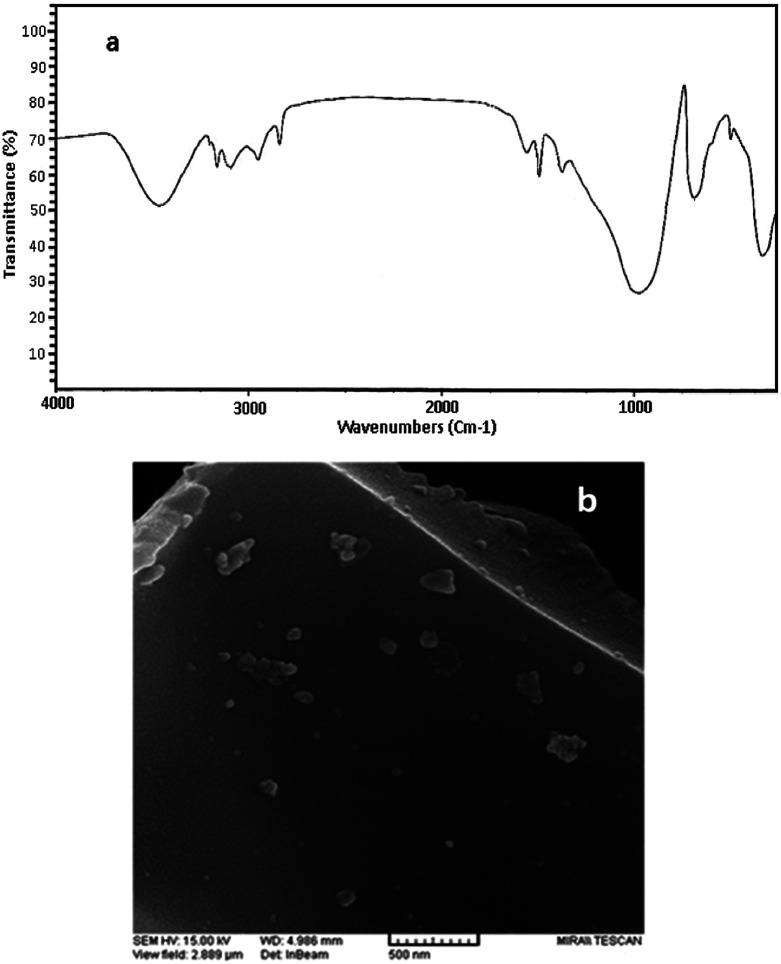
FTIR (a) and SEM image (b) of recovered catalyst.

The characterization of recovered catalyst by FTIR and SEM methods was shown in Fig. 7. As can be seen, no changes were occurred on morphology and particle size of recycled catalyst; also, the main peaks are identical in both of the fresh and recycled catalyst. Consequently, the prepared catalyst has high stability and high efficiency in solvent-free synthesis of xanthenes.

## Experimental

### Materials and apparatus

Chemicals such as dimedone, *N*,*N*-dimethylbarbituric acid, aldehyde derivatives, 2-naphthol, methyl imidazole, perlite nanoparticles, chloropropyltriethoxysilane, zirconium tetrachloride, methanol, organic solvents and hydrochloric acid were purchased from Fluka, Merck and Aldrich chemical companies. All of products were characterized by their spectral data. ^1^H NMR spectra were recorded on a Avance BRUKER (DRX – 400 MHz) in CDCl_3_ or DMSO-d_6_ as solvent. FTIR spectra were determined on a Nicolet Magna series FTIR 550 spectrometer using KBr pellets. Thin layer chromatography (TLC) on commercial aluminium-backed plates of silica gel 60 F_254_ was used to monitor the progress of the reactions. Thermo gravimetric curve of perlite NPs@IL/ZrCl_4_ were obtained from a STA503 analyzer. XRD patterns were collected on a Philips Xpert MPD diffractometer equipped with a Cu Kα anode (*λ* = 1.54 Å) in the 2*θ* range from 10 to 80°. Morphology of perlite NPs and catalyst, were analyzed by SEM using a MIRA/TESCAN with accelerating voltage of 120 kV.

### Preparation of perlite nanoparticles

**Scheme 4:**
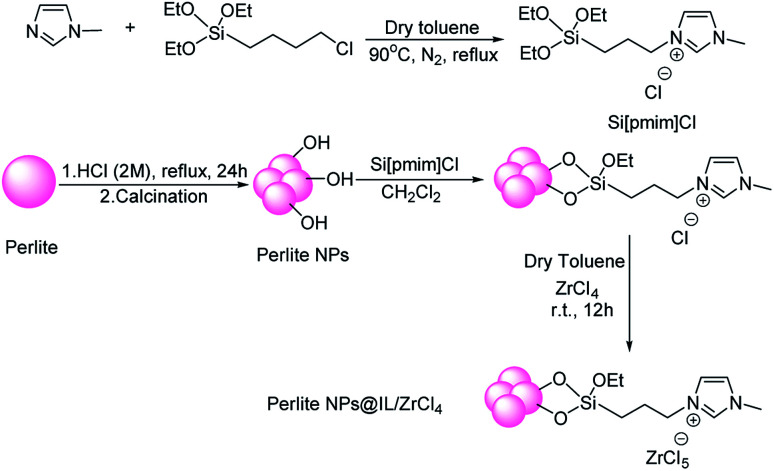
Preparation of perlite NPs@IL/ZrCl_4_.

To make perlite from a crystalline to nanosize structure, 5 g of perlite was heated in 500 °C for 1 hour. Then HCl (150 ml, 2 M) was added and refluxed for 24 hours. After that, the mixture was filtered, washed and pH was controlled to neutralize. Finally, the precipitate was dried in 100 °C for 4 hours and then calcinated in 700 °C for 2 hours^23^ (Scheme 4).

### Preparation of perlite NPs@IL/ZrCl_4_

In first step for preparation of catalyst, 1-(3-triethoxysilyl)-propyl-3-methylimidazolium chloride (Si[pmim]Cl) was synthesized according to Kumar method.^65^ A mixture of methyl imidazole (1 mmol) and 3-chloropropyltriethoxysilane (1 mmol) was refluxed in 5 ml anhydrous toluene for 2 hours under N_2_ atmosphere. After the reaction time, obtained viscous ionic liquid was extracted by diethyl ether. In order to attach the ionic liquid to perlite nanoparticle surfaces, 1 g of perlite nanoparticles was added to 0.6 g of Si[pmim]Cl and stirred for 16 hours at 90 °C. After the time, the mixture was washed with 10 ml of boiling dichloromethane and dried. Then, 0.7 g ZrCl_4_ dissolved in anhydrous toluene was added to obtained precipitate and stirred at room temperature for 12 hours. Finally, prepared catalyst was dried at 80 °C and characterized by FT-IR, XRD, SEM, EDX and TGA techniques (Scheme 2).

### General procedure for the preparation of xanthene derivatives

In order to achieve satisfactory results, in a general procedure, a mixture of 1 mmol benzaldehyde derivative, 2 mmol dimedone or (1 mmol 2-naphthol and 1 mmol dimedone) in the presence of optimized amount of perlite@IL/ZrCl_4_ nanoparticles (0.005 g, 0.4 mol%) was stirred for adequate times under conventional heating conditions at 80 °C. After completion of the reaction, the mixture was resolved in hot ethanol and catalyst was separated by simple filtration. The crude product was obtained after evaporation and for further purification, recrystallized from ethanol. In case of naphthopyranopyrimidines, a mixture of 1 mmol benzaldehyde derivative, 1 mmol dimedone and 1 mmol *N*,*N*-dimethylbarbituric acid was stirred in the presence of 0.005 g, (0.4 mol%) of catalyst at 80 °C. Separation and purification of reaction products was done according to xanthene route.

### Spectral data

#### 9,9-Dimethyl-12-(4-methylphenyl)-8,9,10,12-tetrahydrobenzo[*a*]xanthene-11-one (4a)

White solid. Mp: 205–207 °C. *ν̄*_max_ (KBr)/cm^−1^: 3015 (=C–H), 2958 (–C–H), 1667 (C=O), 1596–1417 (C=C), 1374 (CH_3_, bending), 1300–1000 (C–O), 831 (=C–H bending OOP of *para* disubstituted phenyl ring). ^1^H NMR (DMSO-d_6_)/ppm: *δ*_H_ = 8.01 (1H, d, ^3^*J*_HH_ = 7.6 Hz, Ar–H), 7.49 (2H, d, ^3^*J*_HH_ = 8.4 Hz, Ar–H), 7.55–7.42 (3H, m, Ar–H), 7.15 (2H, d, ^3^*J*_HH_ = 8.0 Hz, Ar–H), 6.96 (2H, d, ^3^*J*_HH_ = 8.0 Hz, Ar–H), 5.50 (1H, s, CH), 2.56 (2H, d, ^2^*J*_HH_ = 17.6 Hz, CH_2_C=O), 2.32 (2H, d, ^2^*J*_HH_ = 16.4 Hz, CH_2_), 2.12 (3H, s, CH_3_), 1.05 (3H, s, CH_3_), 0.88 (3H, s, CH_3_).

#### 9,9-Dimethyl-12-(4-hydroxyphenyl)-8,9,10,12-tetrahydrobenzo[*a*]xanthene-11-one (4b)

Pale yellow solid. Mp: 215–217 °C. *ν̄*_max_ (KBr)/cm^−1^: 3309 (O–H), 3050 (=C–H), 1717 (C=O), 1642–1400 (C=C), 1366 (CH_3_, bending), 1251 (Ar–O), 1300–1000 (C–O), 850 (=C–H bending OOP of *para* disubstituted phenyl ring). ^1^H NMR (DMSO-d_6_)/ppm: *δ*_H_ = 9.20 (1H, s, OH), 8.12 (1H, d, ^3^*J*_HH_ = 9.2 Hz, Ar–H), 7.88 (2H, t, ^3^*J*_HH_ = 9.2 Hz, Ar–H), 7.44–7.32 (3H, m, Ar–H), 7.26 (2H, d, ^3^*J*_HH_ = 8.0 Hz, Ar–H), 6.65 (2H, d, ^3^*J*_HH_ = 8.0 Hz, Ar–H), 5.63 (1H, s, CH), 2.67 (2H, d, ^2^*J*_HH_ = 16.4 Hz, CH_2_C=O), 2.32 (1H, d, ^2^*J*_HH_ = 16.0 Hz, CH_2_), 2.10 (1H, d, ^2^*J*_HH_ = 16.0 Hz, CH_2_), 1.05 (3H, s, CH_3_), 0.88 (3H, s, CH_3_).

#### 9,9-Dimethyl-12-(4-methoxyphenyl)-8,9,10,12-tetrahydrobenzo[*a*]xanthene-11-one (4c)

White solid. Mp: 204–206 °C. *ν̄*_max_ (KBr)/cm^−1^: 3015 (=C–H), 2955 (–C–H), 1648 (C=O), 1597–1462 (C=C), 1377 (CH_3_, bending), 1300–1000 (C–O), 831 (=C–H bending OOP of *para* disubstituted phenyl ring). ^1^H NMR (DMSO-d_6_)/ppm: *δ*_H_ = 8.05 (1H, d, ^3^*J*_HH_ = 7.6 Hz, Ar–H), 7.95 (2H, d, ^3^*J*_HH_ = 8.4 Hz, Ar–H), 7.48–7.38 (3H, m, Ar–H), 7.15 (2H, d, ^3^*J*_HH_ = 8.0 Hz, Ar–H), 6.72 (2H, d, ^3^*J*_HH_ = 8.0 Hz, Ar–H), 5.50 (1H, s, CH), 3.61 (3H, s, OCH_3_), 2.46 (2H, d, ^2^*J*_HH_ = 17.6 Hz, CH_2_C=O), 2.36 (1H, d, ^2^*J*_HH_ = 16.4 Hz, CH_2_), 2.15 (1H, d, ^2^*J*_HH_ = 16.4 Hz, CH_2_), 1.05 (3H, s, CH_3_), 0.90 (3H, s, CH_3_).

#### 9,9-Dimethyl-12-(2-chlorophenyl)-8,9,10,12-tetrahydrobenzo[*a*]xanthene-11-one (4d)

White solid. Mp: 178–180 °C. *ν̄*_max_ (KBr)/cm^−1^: 3061 (=C–H), 2956 (–C–H), 1670 (C=O), 1622–1469 (C=C), 1373 (CH_3_, bending), 1300–1000 (C–O), 741 (=C–H bending OOP of *ortho* disubstituted phenyl ring). ^1^H NMR (DMSO-d_6_)/ppm: *δ*_H_ = 8.12 (1H, t, ^3^*J*_HH_ = 8.0 Hz, Ar–H), 7.90 (2H, d, ^3^*J*_HH_ = 8.0 Hz, Ar–H), 7.67–7.00 (7H, m, Ar–H), 5.78 (1H, s, CH), 2.68–2.67 (4H, m, CH_2_C=O and CH_2_), 1.05 (3H, s, CH_3_), 0.88 (3H, s, CH_3_).

#### 4-(9,9-Dimethyl-11-oxo-8,10,11,12-tetrahydro-9*H*-benzo[*a*]xanthene-12-yl)-benzaldehyde (4e)

White yellow solid. Mp: 306–308 °C. *ν̄*_max_ (KBr)/cm^−1^: 3058 (=C–H), 2958 (–C–H), 1670 (C=O), 1595–1451 (C=C), 1367 (CH_3_, bending), 1300–1000 (C–O). ^1^H NMR (DMSO-d_6_)/ppm: *δ*_H_ = 8.05–7.90 (3H, m, Ar–H), 7.60–7.45 (3H, m, Ar–H), 7.05 (2H, s, Ar–H), 5.58 (1H, s, CH), 2.54–2.00 (4H, m, CH_2_C=O and CH_2_), 1.00 (3H, s, CH_3_), 0.75 (3H, s, CH_3_).

#### 9-(3-Nitrophenyl)-3,3,6,6-tetramethyl-3,4,5,6,7,9-hexahydro-1*H*-xanthene-1,8-(2*H*)-dione (5a)

White solid. Mp: 171–173 °C. *ν̄*_max_ (KBr)/cm^−1^: 3063 (=C–H), 2961 (–C–H), 1662 (C=O), 1623–1429 (C=C), 1355 and 1529 (N=O), 1355 (CH_3_, bending), 1300–1000 (C–O). ^1^H NMR (DMSO-d_6_)/ppm: *δ*_H_ = 7.98–7.97 (2H, m, Ar–H), 7.64 (1H, d, ^3^*J*_HH_ = 8.0 Hz, Ar–H), 7.55 (1H, t, ^3^*J*_HH_ = 8.0 Hz, Ar–H), 4.65 (1H, s, CH), 2.58–2.48 (4H, m, CH_2_C=O), 2.27 (2H, d, ^2^*J*_HH_ = 16.0 Hz, CH_2_), 2.10 (2H, d, ^2^*J*_HH_ = 16.0 Hz, CH_2_), 1.03 (6H, s, CH_3_), 0.89 (6H, s, CH_3_).

#### 9-(2,4-Dichlorophenyl)-3,3,6,6-tetramethyl-3,4,5,6,7,9-hexahydro-1*H*-xanthene-1,8-(2*H*)-dione (5b)

White solid. Mp: 245–247 °C. *ν̄*_max_ (KBr)/cm^−1^: 3070 (=C–H), 2962 (–C–H), 1661 (C=O), 1623–1424 (C=C), 1359 (CH_3_, bending), 1300–1000 (C–O). ^1^H NMR (DMSO-d_6_)/ppm: *δ*_H_ = 7.40 (1H, s, Ar–H), 7.28 (2H, m, Ar–H), 4.75 (1H, s, CH), 2.57 (2H, d, ^2^*J*_HH_ = 18.4 Hz, CH_2_C=O), 2.46 (2H, d, ^2^*J*_HH_ = 18.4 Hz, CH_2_C=O), 2.27 (2H, d, ^2^*J*_HH_ = 16.0 Hz, CH_2_), 2.05 (2H, d, ^2^*J*_HH_ = 16.0 Hz, CH_2_), 1.02 (6H, s, CH_3_), 0.90 (6H, s, CH_3_).

#### 9-(3-Methoxyphenyl)-3,3,6,6-tetramethyl-3,4,5,6,7,9-hexahydro-1*H*-xanthene-1,8-(2*H*)-dione (5c)

White solid. Mp: 240–242 °C. *ν̄*_max_ (KBr)/cm^−1^: 3050 (=C–H), 2958 (–C–H), 1662 (C=O), 1592–1489 (C=C), 1370 (CH_3_, bending), 1300–1000 (C–O), 824 (=C–H bending OOP of *para* disubstituted phenyl ring). ^1^H NMR (DMSO-d_6_)/ppm: *δ*_H_ = 7.04 (2H, d, ^3^*J*_HH_ = 6.4 Hz, Ar–H), 6.75 (2H, d, ^3^*J*_HH_ = 7.2 Hz, Ar–H), 4.45 (1H, s, CH), 3.66 (3H, s, OCH_3_), 2.69–2.63 (4H, m, CH_2_C=O), 2.23 (2H, d, ^2^*J*_HH_ = 16.0 Hz, CH_2_), 2.07 (2H, d, ^2^*J*_HH_ = 16.0 Hz, CH_2_), 1.02 (6H, s, CH_3_), 0.88 (6H, s, CH_3_).

#### 9-(4-Chlorophenyl)-3,3,6,6-tetramethyl-3,4,5,6,7,9-hexahydro-1*H*-xanthene-1,8-(2*H*)-dione (5d)

White solid. Mp: 225–227 °C. *ν̄*_max_ (KBr)/cm^−1^: 3050 (=C–H), 2957 (–C–H), 1663 (C=O), 1594–1485 (C=C), 1364 (CH_3_, bending), 1300–1000 (C–O), 742 (=C–H bending OOP of *ortho* disubstituted phenyl ring). ^1^H NMR (DMSO-d_6_)/ppm: *δ*_H_ = 7.26–7.12 (3H, m, Ar–H), 7.09 (1H, t, ^3^*J*_HH_ = 5.6 Hz, Ar–H), 4.80 (1H, s, CH), 2.62 (2H, d, ^2^*J*_HH_ = 17.6 Hz, CH_2_C=O), 2.48 (2H, d, ^2^*J*_HH_ = 17.6 Hz, CH_2_C=O), 2.24 (2H, d, ^2^*J*_HH_ = 16.4 Hz, CH_2_), 2.02 (2H, d, ^2^*J*_HH_ = 16.4 Hz, CH_2_), 1.02 (6H, s, CH_3_), 0.90 (6H, s, CH_3_).

#### 4,4′-Benzilidine-bis[3,3,6,6-tetramethyl-3,4,5,6,7,9-hexahydro-1*H*-xanthene-1,8-(2*H*)-dione] (5e)

White solid. Mp: 228–230 °C. *ν̄*_max_ (KBr)/cm^−1^: 3049 (=C–H), 2962 (–C–H), 1610 (C=O), 1584–1400 (C=C), 1389 (CH_3_, bending), 1300–1000 (C–O), 828 (=C–H bending OOP of *para* disubstituted phenyl ring). ^1^H NMR (DMSO-d_6_)/ppm: *δ*_H_ = 7.40 (4H, s, Ar–H), 4.75 (2H, s, CH), 2.77–2.49 (8H, m, CH_2_C=O), 2.26 (4H, d, ^2^*J*_HH_ = 16.0 Hz, CH_2_), 2.06 (4H, d, ^2^*J*_HH_ = 16.0 Hz, CH_2_), 1.02 (12H, s, CH_3_), 0.88 (12H, s, CH_3_).

#### 12-(4-Hydroxyphenyl)-8,10-dimethyl-8,10-dihydro-9*H*-benzo[5,6]chromene[2,3,*d*]pyrimidine-9,11-(10*H*)-dione (7a)

Cream solid. Mp: 286–288 °C. *ν̄*_max_ (KBr)/cm^−1^: 3206 (O–H), 3053 (=C–H), 2926 (–C–H), 1669 (C=O), 1541–1417 (C=C), 1356 (CH_3_, bending), 1300–1000 (C–O), 855 (=C–H bending OOP of *para* disubstituted phenyl ring). ^1^H NMR (DMSO-d_6_)/ppm: *δ*_H_ = 8.90 (1H, s, OH), 8.30 (2H, d, ^3^*J*_HH_ = 8.0 Hz, Ar–H), 8.03–7.85 (3H, m, Ar–H), 7.53–7.43 (3H, m, Ar–H), 6.95 (2H, d, ^3^*J*_HH_ = 8.0 Hz, Ar–H), 5.98 (1H, s, CH), 3.33 (3H, s, CH_3_), 3.19 (3H, s, CH_3_).

#### 12-(2,4-Dichlorophenyl)-8,10-dimethyl-8,10-dihydro-9*H*-benzo[5,6]chromene[2,3,*d*]pyrimidine-9,11-(10*H*)-dione (7b)

White solid. Mp: 263–265 °C. *ν̄*_max_ (KBr)/cm^−1^: 3058 (=C–H), 2952 (–C–H), 1707 (C=O), 1661–1456 (C=C), 1355 (CH_3_, bending), 1300–1000 (C–O). ^1^H NMR (DMSO-d_6_)/ppm: *δ*_H_ = 7.55–7.42 (4H, m, Ar–H), 7.31 (1H, s, Ar–H), 7.12 (1H, d, ^3^*J*_HH_ = 9.6 Hz, Ar–H), 6.90–6.65 (3H, m, Ar–H), 5.77 (1H, s, CH), 3.50 (3H, s, CH_3_), 3.20 (3H, s, CH_3_).

#### 12-(4-Nitrophenyl)-8,10-dimethyl-8,10-dihydro-9*H*-benzo[5,6]chromene[2,3,*d*]pyrimidine-9,11-(10*H*)-dione (7c)

Cream solid. Mp: 286–288 °C. *ν̄*_max_ (KBr)/cm^−1^: 3090 (=C–H), 2850 (–C–H), 1753 (C=O), 1674–1441 (C=C), 1575 and 1342 (N=O), 1342 (CH_3_, bending), 1300–1000 (C–O), 836 (=C–H bending OOP of *para* disubstituted phenyl ring). ^1^H NMR (DMSO-d_6_)/ppm: *δ*_H_ = 8.05–7.90 (5H, m, Ar–H), 7.68–7.63 (2H, m, Ar–H), 7.55–7.44 (3H, m, Ar–H), 5.83 (1H, s, CH), 3.49 (3H, s, CH_3_), 3.13 (3H, s, CH_3_).

#### 12-(4-Methylphenyl)-8,10-dimethyl-8,10-dihydro-9*H*-benzo[5,6]chromene[2,3,*d*]pyrimidine-9,11-(10*H*)-dione (7d)

White solid. Mp: 195–197 °C. *ν̄*_max_ (KBr)/cm^−1^: 3015 (=C–H), 2952 (–C–H), 1709 (C=O), 1630–1452 (C=C), 1399 (CH_3_, bending), 1300–1000 (C–O), 808 (=C–H bending OOP of *para* disubstituted phenyl ring). ^1^H NMR (DMSO-d_6_)/ppm: *δ*_H_ = 8.03–7.90 (3H, m, Ar–H), 7.59 (1H, t, ^3^*J*_HH_ = 8.0 Hz, Ar–H), 7.56–7.48 (2H, m, Ar–H), 7.19 (2H, d, ^3^*J*_HH_ = 6.8 Hz, Ar–H), 6.97 (2H, d, ^3^*J*_HH_ = 6.8 Hz, Ar–H), 5.60 (1H, s, CH), 3.49 (3H, s, CH_3_), 3.14 (3H, s, CH_3_), 2.12 (3H, s, CH_3_).

#### 12-(4-Methoxyphenyl)-8,10-dimethyl-8,10-dihydro-9*H*-benzo[5,6]chromene[2,3,*d*]pyrimidine-9,11-(10*H*)-dione (7e)

Yellow solid. Mp: 292–294 °C. *ν̄*_max_ (KBr)/cm^−1^: 3103 (=C–H), 2955 (–C–H), 1737 (C=O), 1665–1432 (C=C), 1362 (CH_3_, bending), 1300–1000 (C–O), 1267 and 1084 (Ar–C–O), 849 (=C–H bending OOP of *para* disubstituted phenyl ring). ^1^H NMR (DMSO-d_6_)/ppm: *δ*_H_ = 8.05–7.67 (3H, m, Ar–H), 7.55–7.44 (4H, m, Ar–H), 7.20 (2H, d, ^3^*J*_HH_ = 7.2 Hz, Ar–H), 6.67 (2H, d, ^3^*J*_HH_ = 7.2 Hz, Ar–H), 5.60 (1H, s, CH), 3.49 (3H, s, CH_3_), 3.35 (3H, s, OCH_3_), 3.21 (3H, s, CH_3_).

#### 12-(2-Hydroxyphenyl)-8,10-dimethyl-8,10-dihydro-9*H*-benzo[5,6]chromene[2,3,*d*]pyrimidine-9,11-(10*H*)-dione (7f)

Cream solid. Mp: 288–290 °C. *ν̄*_max_ (KBr)/cm^−1^: 3300 (O–H), 3052 (=C–H), 2962 (–C–H), 1700 (C=O), 1584–1400 (C=C), 1379 (CH_3_, bending), 1300–1000 (C–O), 759 (=C–H bending OOP of *ortho* disubstituted phenyl ring). ^1^H NMR (DMSO-d_6_)/ppm: *δ*_H_ = 8.75 (1H, s, OH), 7.78–7.52 (3H, m, Ar–H), 7.32–6.85 (7H, m, Ar–H), 5.83 (1H, s, CH), 3.49 (3H, s, CH_3_), 3.13 (3H, s, CH_3_).

#### 12-(3-Methoxyphenyl)-8,10-dimethyl-8,10-dihydro-9*H*-benzo[5,6]chromene[2,3,*d*]pyrimidine-9,11-(10*H*)-dione (7g)

Yellow solid. Mp: 283–285 °C. *ν̄*_max_ (KBr)/cm^−1^: 3027 (=C–H), 2959 (–C–H), 1671 (C=O), 1595–1451 (C=C), 1368 (CH_3_, bending), 1300–1000 (C–O). ^1^H NMR (DMSO-d_6_)/ppm: *δ*_H_ = 7.65 (2H, t, ^3^*J*_HH_ = 7.6 Hz, Ar–H), 7.55–7.42 (5H, m, Ar–H), 7.29–7.07 (3H, m, Ar–H), 5.87 (1H, s, CH), 3.65 (3H, s, OCH_3_), 3.56 (3H, s, CH_3_), 3.12 (3H, s, CH_3_).

## Conclusions

In this study, perlite nanoparticles was prepared and modified with Lewis acidic ionic liquid (perlite NPs@IL/ZrCl_4_) and characterized with FTIR, XRD, SEM, EDX and TGA techniques. We presented a new efficient and environmental friendly pathway for one pot synthesis of xanthene and naphthopyranopyrimidine derivatives *via* multicomponent reactions using perlite NPs@IL/ZrCl_4_ as a recyclable effective solid acid catalyst. This simple procedure is solvent free and it's easy and clean work up, high yield of products and reusability of catalyst as well as low cost and very little amounts of catalyst (0.005 g, 0.4 mol%), make this method considerable for the synthesis of other organic compounds.

## Conflicts of interest

There are no conflicts to declare.

## Supplementary Material

## References

[cit1] El-Brashy M., Metwally M. E., El-Sepai F. A. (2004). IL Farmaco.

[cit2] Chibale K., Visser M., Schalkwyk D. V., Smith P. J., Saravanamuthu A., Fairlamb A. H. (2003). Tetrahedron.

[cit3] Bhowmik B. B., Ganguly P. (2005). Spectrochim. Acta, Part A.

[cit4] Ahmad M., King T. A., Ko D. K., Cha B. H., Lee J. (2002). J. Phys. D: Appl. Phys..

[cit5] Knight C. G., Stephens T. (1989). Biochem. J..

[cit6] Eid F. A., Abd El-Waheb A. H. F., El-Hag Ali G. A. M., Khafagy M. M. (2004). Acta Pharm..

[cit7] Bedair A. H., El-Hady N. A., El-Latif M. S. A., Fakery A. H., El-Agrody A. M. (2000). IL Farmaco.

[cit8] Bruno O., Brullo C., Schenone S., Bondavalli F., Ranise A., Tognolini M. (2006). Bioorg. Med. Chem..

[cit9] Joshi K. C., Jain R., Sharma K., Bhattacharya S. K., Goel R. K. (1988). J. Indian Chem. Soc..

[cit10] Bruno O., Brullo C., Schenone S., Ranise A., Bondavalli F., Barocelli E., Tognolini M., Magnanini F., Ballabeni V. (2002). IL Farmaco.

[cit11] Bruno O., Brullo C., Schenone S., Bondavalli F., Ranise A., Tognolini M., Ballabeni V., Barocelli E. (2004). Bioorg. Med. Chem..

[cit12] Chabchoub F., Messaâd M., Ben Mansour H., Ghdira L., Salem M. (2007). Eur. J. Med. Chem..

[cit13] Khafagy M. M., Abd El-Wahab A. H. F., Eid F. A., El-Agrody A. M. (2002). IL Farmaco.

[cit14] Messaâd M., Chabchoub F., Salem M. (2005). Heterocycl. Commun..

[cit15] Kodama O., Ichikawa H., Akatsuka T., Santisopasri V., Kato A., Hayashi Y. (1993). J. Nat. Prod..

[cit16] Mdhav J. V., Kuram B. S., Rajitha B. (2008). ARKIVOC.

[cit17] Das B., Ravikanth B., Ramu R., Laxminarayana K., Rao B. v. (2006). J. Mol. Catal. A: Chem..

[cit18] Liu P., Jian-Wu H., Liang S. J., Liang G. L., Wang J. Y., Zhang Z.-H. (2016). Monatsh. Chem..

[cit19] Karami B., Eskandari K., Zare Z., Gholipour S. (2014). Chem. Heterocycl. Compd..

[cit20] Safaei-Ghomi J., Ghasemzadeh M. A. (2012). Chin. Chem. Lett..

[cit21] Shaterian H. R., Ghashang M., Mir N. (2007). ARKIVOC.

[cit22] Rajitha B., Kumar B. S., Reddy B. Y. T., Reddy P. N. (2005). Tetrahedron Lett..

[cit23] Mirhadi E., Ramazani A., Rouhani M., Woo Joo S. (2013). Chemija.

[cit24] Saini A., Kumar S., Sandhu J. S. (2006). Synlett.

[cit25] Seyyedhamzeh M., Mirzaei P., Bazgir A. (2008). Dyes Pigm..

[cit26] Kantevari S., Chary M. V., Das A. P. R., Vuppalapati S. V. N., Lingaiah N. (2008). Catal. Commun..

[cit27] Ohishi T., Kojima T., Matsuoka T., Shiro M., Kotsuki H. (2001). Tetrahedron Lett..

[cit28] Rao Chinta R., Harikrishna V., Kumar Tulam V., Mainkar P. S., Dubey P. K. (2016). Asian J. Chem..

[cit29] Sun X. J., Zhou J. F., Zhao P. S. (2011). J. Heterocycl. Chem..

[cit30] Jalde S. S., Chavan H. V., Adsul L. K., Dhakane V. D., Bandgar B. P. (2014). Synth. React. Inorg., Met.-Org., Nano-Met. Chem..

[cit31] Shaterian H. R., Azizi K., Fahimi N. (2014). Res. Chem. Intermed..

[cit32] Lakouraj M. M., Fallah Z., Tajbakhsh M., Hasantabar V. (2014). Caspian Journal of Chemistry.

[cit33] Naeimi H., Nazifi Z. S. (2013). J. Chin. Chem. Soc..

[cit34] Hajipour A. R., Ghayeb Y., Sheikhan N., Ruoho A. E. (2010). Synlett.

[cit35] Gong K., Fang D., Wang H.-L., Zhou X.-L., Liu Z.-L. (2009). Dyes Pigm..

[cit36] Zhu A., Bai S., Jin W., Liu R., Li L., Zhao Y., Wang J. (2014). RSC Adv..

[cit37] Yang J., Yang J., Zhu T., Wang P., Fang D. (2013). Monatsh. Chem..

[cit38] Kumari P., Yathindranath V., Chauhan S. M. S. (2008). Synth. Commun..

[cit39] Arifuzzaman M., Kim H. S. (2017). Constr. Build. Mater..

[cit40] Singh M., Garg M. (1991). Constr. Build. Mater..

[cit41] Arifuzzaman M., Kim H. S. (2017). Constr. Build. Mater..

[cit42] Shastri D., Kim H. S. (2014). Constr. Build. Mater..

[cit43] Torabi S. F., Khajeh K., Ghasempur S., Ghaemi N., Siadat S. O. R. (2007). J. Biotechnol..

[cit44] Rostami-Vartooni A., Nasrollahzadeh M., Alizadeh M., Hölderich W. F. (2016). J. Alloys Compd..

[cit45] Maleki B., Gholizadeh M., Sepehr Z. (2011). Bull. Korean Chem. Soc..

[cit46] Azimi S. C. (2015). Iran. J. Catal..

[cit47] Nandi G. C., Samai S., Singh M. S. (2010). Synlett.

[cit48] Mohaqeq M., Safaei-Ghomi J., Shahbazi-Alavi H., Teymuri R. (2017). Polycyclic Aromat. Compd..

[cit49] Das P. J., Das J. (2015). RSC Adv..

[cit50] Ghaffari Khaligh N. (2018). Res. Chem. Intermed..

[cit51] Chandra N. G., Subhasis S., Kumar R., Singh M. S. (2009). Tetrahedron.

[cit52] Aayesha N. (2013). Asian J. Chem..

[cit53] Niknam K., Damya M. (2009). J. Chin. Chem. Soc..

[cit54] Dadhania A. N., Patel V. K., Raval D. K. (2012). C. R. Chim..

[cit55] Eshghi H., Rahimizadeh M., Eftekhar M., Bakavoli M. (2014). Kinet. Catal..

[cit56] Sajadi S. M., Maham M., Ahmad B. O. (2014). Lett. Org. Chem..

[cit57] Soleimani O., Hosseinian A. (2018). J. Chem. Res..

[cit58] Rahmati A. (2010). Chin. Chem. Lett..

[cit59] Zare A., Moosavi-Zare A. R., Merajoddin M., Zolfigol M. A., Hekmat-Zadeh T., Hasaninejad A., Khazaei A., Mokhlesi M., Khakyzadeh V., Derakhshan-Panah F., Beyzavi M. H., Rostami E., Arghoon A., Roohandeh R. (2012). J. Mol. Liq..

[cit60] Rad-Moghadam K., Azimi S. C. (2012). J. Mol. Catal. A: Chem..

[cit61] Sajadikhah S. S. (2015). RSC Adv..

[cit62] Jalde S. S., Chavan H. V., Adsul L. K., Dhakane V. D., Bandgar B. P. (2014). Synth. React. Inorg. Met.-Org. Chem..

[cit63] Praveen Kumar K., Satyanarayana S., Lakshmi Reddy P., Narasimhulu G., Ravirala N., Subba Reddy B. V. (2012). Tetrahedron Lett..

[cit64] Wu L., Wang X., Yang L., Yan F. (2010). Asian J. Chem..

[cit65] Kumar P., Vermeiren W., Dath J. P., Hölderich W. F. (2006). Appl. Catal., A.

